# Engineer design process assisted by explainable deep learning network

**DOI:** 10.1038/s41598-021-01937-5

**Published:** 2021-11-18

**Authors:** Chia-Wei Hsu, An-Cheng Yang, Pei-Ching Kung, Nien-Ti Tsou, Nan-Yow Chen

**Affiliations:** 1 National Yang Ming Chiao Tung University, Hsinchu, Taiwan; 2 National Center for High-Performance Computing, Hsinchu, Taiwan

**Keywords:** Computational methods, Computational science, Biomedical engineering, Mechanical engineering

## Abstract

Engineering simulation accelerates the development of reliable and repeatable design processes in various domains. However, the computing resource consumption is dramatically raised in the whole development processes. Making the most of these simulation data becomes more and more important in modern industrial product design. In the present study, we proposed a workflow comprised of a series of machine learning algorithms (mainly deep neuron networks) to be an alternative to the numerical simulation. We have applied the workflow to the field of dental implant design process. The process is based on a complex, time-dependent, multi-physical biomechanical theory, known as mechano-regulatory method. It has been used to evaluate the performance of dental implants and to assess the tissue recovery after the oral surgery procedures. We provided a deep learning network (DLN) with calibrated simulation data that came from different simulation conditions with experimental verification. The DLN achieves nearly exact result of simulated bone healing history around implants. The correlation of the predicted essential physical properties of surrounding bones (e.g. strain and fluid velocity) and performance indexes of implants (e.g. bone area and bone-implant contact) were greater than 0.980 and 0.947, respectively. The testing AUC values for the classification of each tissue phenotype were ranging from 0.90 to 0.99. The DLN reduced hours of simulation time to seconds. Moreover, our DLN is explainable via Deep Taylor decomposition, suggesting that the transverse fluid velocity, upper and lower parts of dental implants are the keys that influence bone healing and the distribution of tissue phenotypes the most. Many examples of commercial dental implants with designs which follow these design strategies can be found. This work demonstrates that DLN with proper network design is capable to replace complex, time-dependent, multi-physical models/theories, as well as to reveal the underlying features without prior professional knowledge.

## Introduction

Development of devices in various domains requires reliable and repeatable design processes. The process is typically based on trial-and-error in experiments. However, this usually takes many iterations, cost, time, and most importantly, the prior professional knowledge/experience about the particular devices. These facts limit the customization and the advance of the modern devices. Thus, in the recent decades, engineering simulations or virtual experiments^1^ have been widely used to accelerate the design processes. Their advantage is relatively faster and more economical than experiments in the respect of looping through the complete sets of design parameters. However, this leads to the other problems. For example, cumbersome computation is required for the complex design problems; it is still not straight forward to pinpoint the crucial design parameters which dominate the simulation results without the sense of experts.

Recently, machine learning, especially deep learning (DL), has been validated to be a powerful tools in many applications^2,3^ and shown outperformance than professions, such as AlphaGo in board game Go^4^ pediatric bone age assessment^5^, etc. Unlike well-established applications in natural language processing or image/speech recognition, researchers in individual domain are still seeking the opportunity to apply machine learning to their research topics. Jean Rabault et. al.^6^ used deep reinforcement learning network in a flow control problem. In their study, experimental data is used to train a model to perform active flow control with strategy planning. In the domain of atomic simulation, Behler and Parrinello^7^ used neural network to represent the density functional theory (DFT) potential-energy landscape. First principle calculation results were used in training a neuron network regressor, which is an alternate to DFT engine.

Machine learning provides high level of assistance to solve real physical problems and to accelerate engineering design process.

Several successful examples have been proposed in the literature, demonstrating the power of DL serving as an alternative to the specific physical numerical model. For example, Andrew Senior et. al. proposed an advanced protein-structure prediction system, named Alphafold^8^. They trained a neuron network to predict the distances between pairs of residues in protein, and then used the resulting information to construct a mean force potential to describe the structure of protein. Alphafold achieved the highest score in Critical Assessment of protein Structure Prediction (CASP) and has been a well-accepted model in this field. Another successful story is a grand challenge in computational chemistry. The solution of electronic Schrödinger equation of atomic system, which can be solved by Quantum Monte Carlo (QMC), provides most of its chemical properties. However, the computational demands grow exponentially as the increasing of number of electrons and the accuracy is limited by the wavefunction ansatz. Jan Hermann et. al. designed a deep learning network based model: PauliNet^9^ that delivers flexible, physical valid wavefunction ansatz for QMC. PauliNet outperforms conventional ansatzes for molecules with up to 30 electrons with high accuracy compared with it of QMC method while maintaining high computational efficiency. These cases demonstrated the possibility of using multiple networks to be an alternate solution for conventional numerical methods. However, based on the authors’ survey, a very complex, time-dependent, multi-physical models/theories, such as the theories related to biomechanics, have not yet been widely addressed in the literature.

Mechano-regulatory method has been a widely accepted theory for simulating bone healing and tissue regeneration, and applied to the design of dental implants^10^. Two biophysical stimuli: strain and fluid velocity in the bone healing region calculated by finite element (FE) method were considered^11^. The model can predict the distribution of the tissue phenotypes, such as resorption, mature bone, immature bone, cartilage, and fibrous tissue on each day during the healing period, which is difficult to be revealed experimentally. This enables the preoperative evaluations for the dental implants, as well as reduces sacrifices of animals. Although mechano-regulatory method can provide accurate predictions, it still requires hours of calculation and huge resource of computing power, which may not fulfill the demand of efficiency in clinical surgery. Also, the method provides no direct suggestion on the geometry design, and thus, clinic expertise and professional knowledge about dental surgery are still needed.

In this work, we aim to demonstrate that, with the help of a proper designed deep learning network (DLN), a very complex biomechanics model can be replaced and the corresponding computational efficiency can be enhanced. Data of bone healing history around 65 dental implants were generated by mechano-regulatory method and used to train the DLN models. Deep Taylor decomposition was then applied to the portion of DLN, revealing the strategy of implant design, as well as the relevance between physical properties and the bone healing/tissue phenotype regeneration. The contribution of this work is to provide a proof of concept that deep learning approaches can be used as a highly-complex model substitute in on-fly inference, and most importantly, provide in-depth insight with no prior professional knowledge in dental clinical expertise.

## Results

### Network overview

**Figure 1:**
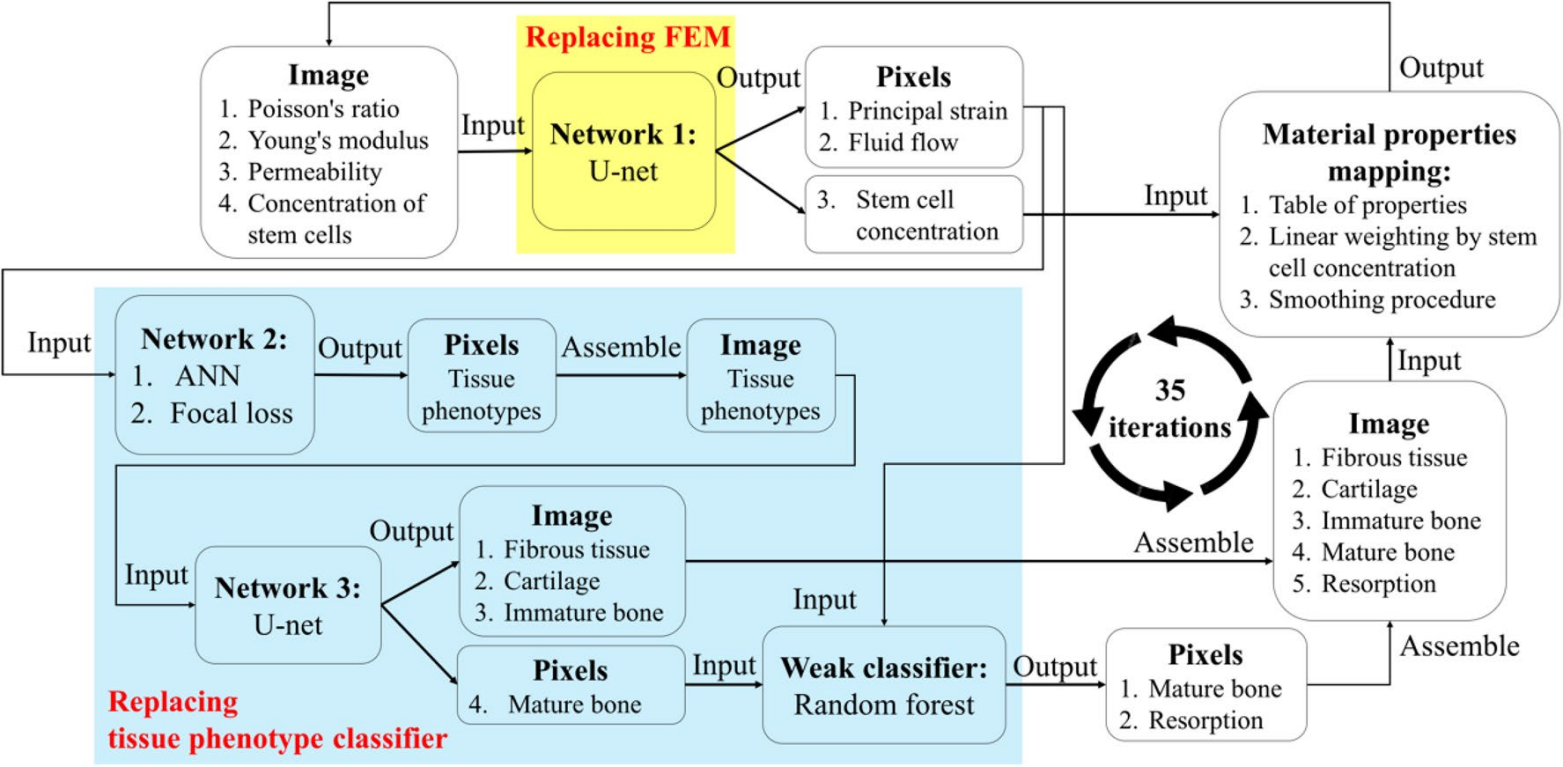
The current DLN structure.

The current network consisted of three neural networks. The detailed workflow is illustrated in Fig. 1. It is worth noting that the reasons why the current work chose this multi-model pipeline over a single End-to-End model are explained in Supplementary. Network 1 (modified from U-Net^12^ and Inception block^13^) was used to replace the mechanical and fluid FE simulation procedures (see “Methods” for more details). The input of Network 1 is the whole image of the implant model with the essential properties, i.e., a matrix with the dimension of 210 × 52 × 4, where the first two dimensions represented the relative position of the implant and the surrounding bones, while the third dimension represented the channels of corresponding material properties, i.e., Young’s modulus, Poisson’s ratio, permeability and the concentration of stem cells. The output was a matrix with the dimension of 210 × 52 × 6, where the third dimension consisted of the predicted principle strain I, II, and III, the fluid velocity in the x and y directions in the current iteration, and stem cell concentration in the next iteration.

Network 2 employed an artificial neural network (ANN) structure, which was used to predict the tissue phenotype at each pixel in the next iteration. This prediction required the 5 outputs from Network 1, apart from stem cell concentration. The output from Network 1 was reformed to 10,920 vectors with the dimension of 1 × 5. Each of which contained the local properties at the corresponding pixel in the image, and was then fed into Network 2. Note that typically, the amount of the data of mature bone was significantly greater than it of resorption. Thus, the focal loss function^14^ was used to reduce the effect of such data imbalance, where the function parameters were determined by Bayesian optimization with tree parzen estimator^15^. Then, the distribution of the tissue phenotypes in the image in the next iteration was obtained by assembling the data of each pixel output from Network 2. However, with the increased iteration, the distribution may contain several singular pixels with their tissue phenotypes different from their neighboring pixels, which may not occur in the results generated by mechano-regulatory method. This error may accumulate along with the iterations. Thus, an additional network was necessary to correct these singular pixels.

While Network 2 performed pixel-wisely, Network 3 (modified from U-Net^12^) performed a structured learning, which deals with the whole image, including singular pixels obtained from Network 2 as the input data. The data for the current iteration generated by mechano-regulatory method was used to train Network 3 to correct the singular pixels. In this way, the number of singular pixels can be reduced, and the accuracy of the prediction throughout the iterations was significantly improved.

By using Networks 1–3, the distribution of the tissue phenotypes around the implant can be predicted. However, in addition to the tissue phenotypes imbalance problem, we found that resorption type was classified incorrectly as mature bone quite frequently. It was due to the small difference between the values of their cell stimulus factor^16^. In order to reduce such type II error of the resorption, the random forest algorithm was applied as a weak classifier. The performance of the classification was improved since the classification domain was reduced to two types in this step.

### The performance of the regression of physical properties

**Figure 2:**
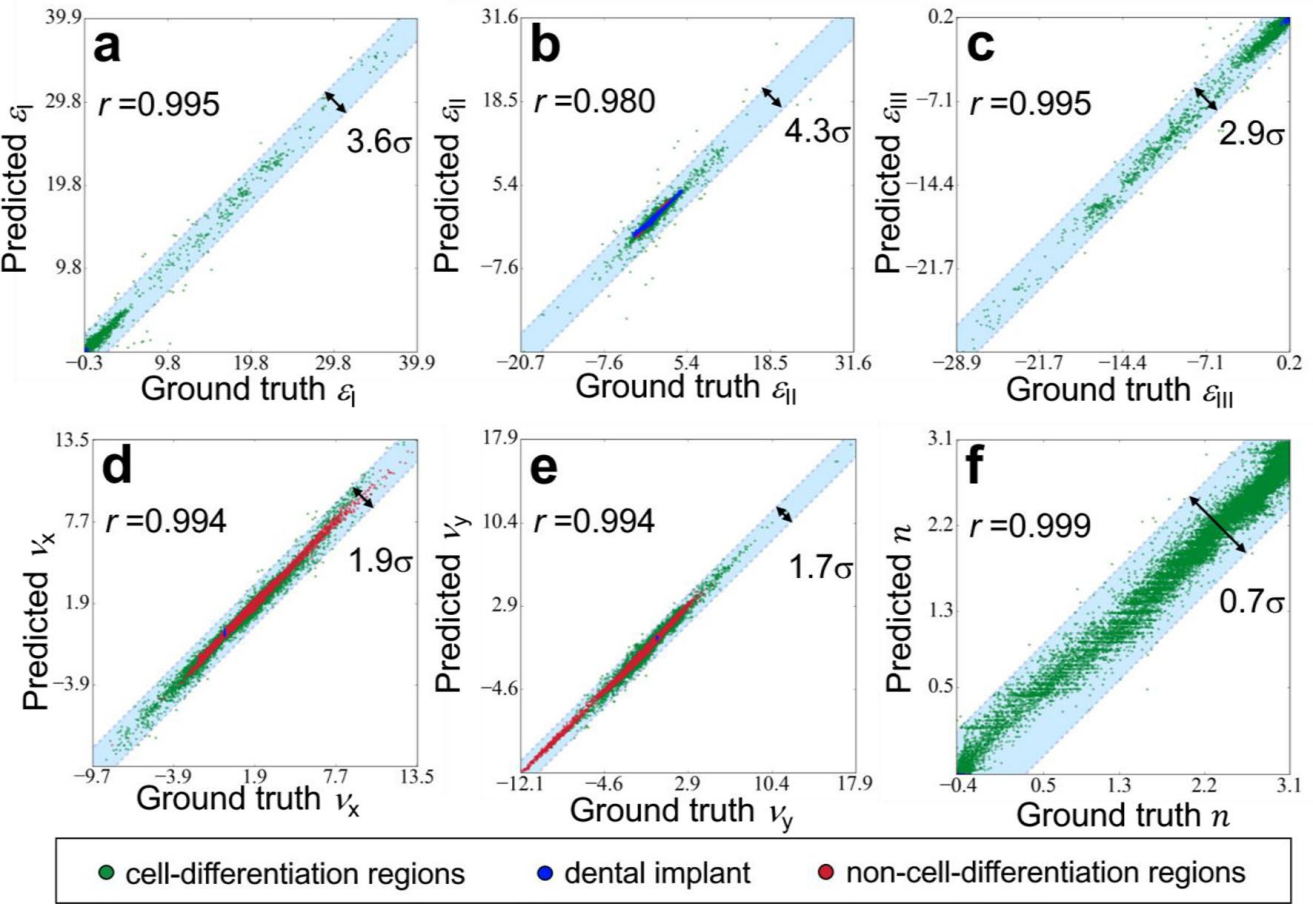
The predicted values vs. ground truth of the six physical properties, principle strain I (*ε*_I_), II (*ε*_II_), and III (*ε*_III_), the fluid velocity in the x and y directions (*ν*_x_, *ν*_y_) and stem cell concentration (*n*), for every pixel throughout 35 days in the 14 implants. Where blue, red, and green dots represented the data from pixels located in dental implant, non-cell-differentiation, and cell-differentiation regions, respectively.

In the current DLN, Network 1 can predict six physical properties, which were determined initially by FE simulation: principle strain I (ε_I_), II (ε_II_), and III (ε_III_), the fluid velocity in the x and y directions (ν_x_, ν_y_) and stem cell concentration (*n*). In order to illustrate the overall view of the results, the predicted values vs. the ground truth of the six physical properties for every pixel throughout 35 days in 14 implant cases (which were randomly chosen among the 65 cases) are shown in Fig. 2 (5,350,800 data points in total). It can be observed that the Pearson correlation coefficients (*r*) of all the six properties were greater than 0.980. The distribution of the scattered data also revealed that there was no significant bias, and even the data with extreme values can be well predicted. Note that the blue, red, and green dots represented the data from pixels located in the dental implant, non-cell-differentiation, and cell-differentiation regions, respectively. Where the pixels of the dental implant (blue dots) associated with strain only, and thus, the corresponding values of flow ν_x_ and ν_y_ accumulated around zero. This demonstrated that the current DLN accurately captured this feature. It is also worth noting that, since the material properties of the pixels in dental implant and non-cell-differentiation regions (blue and red dots) remained constant throughout 35 days, their data can be predicted with higher accuracy compared to those in the cell-differentiation region (green dots). The light blue region in each plot shown in Fig. 2 contained 99.99% data points; the double arrows indicated the width of the blue region with the unit of the standard deviation σ. The results show the excellent regression performance of Network 1.

### The performance of classification of tissue phenotypes

**Figure 3:**
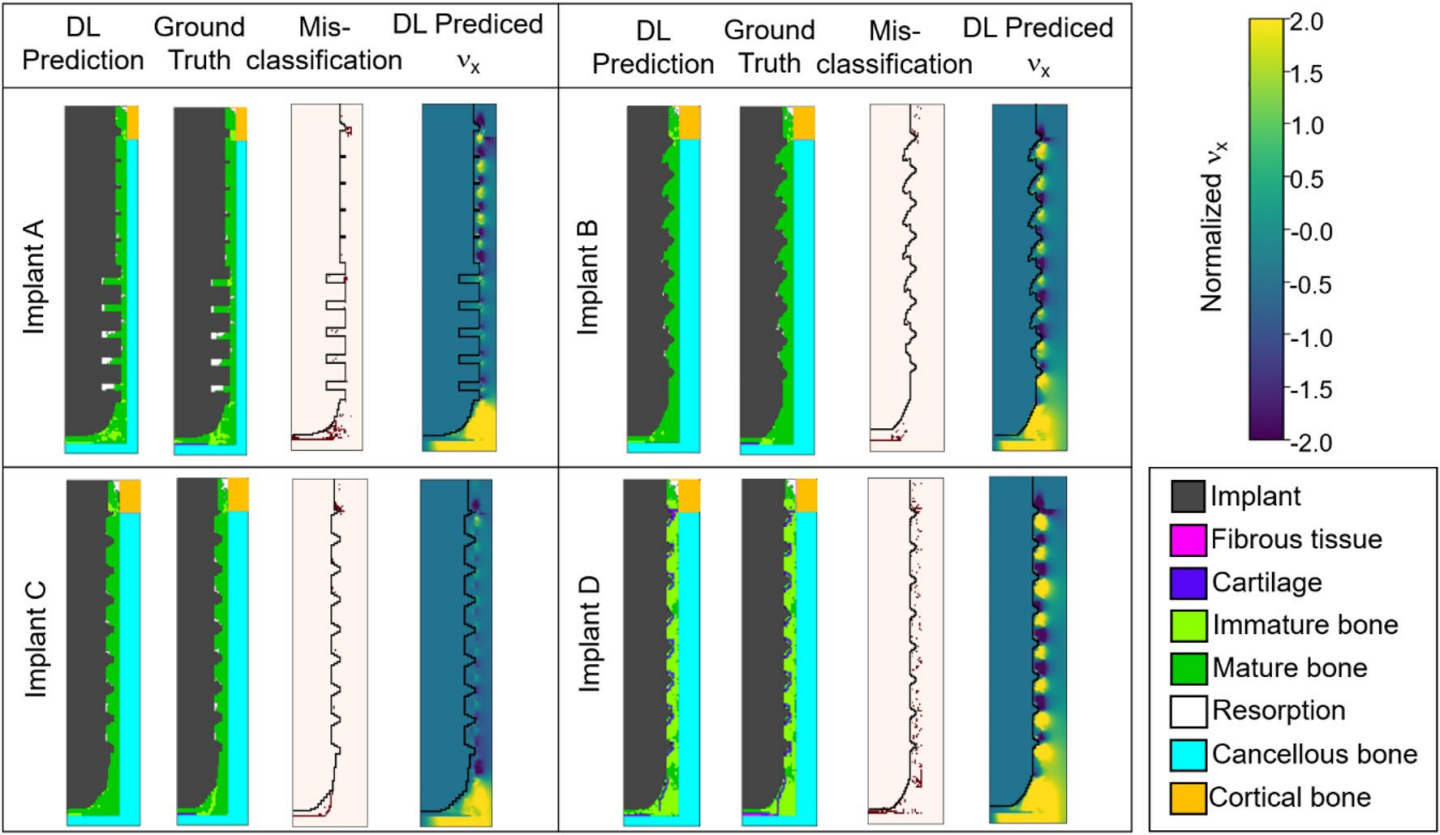
The distribution of tissue phenotypes predicted by the DLN, the ground truth generated by mechano-regulatory method, misclassification, and DL predicted *ν*_*x*_.

Four implants, referred as Implant A to D, among the all the dataset of 65 implants were chosen to demonstrate the validity of the current DLN. The corresponding distributions of tissue phenotype around the four implants on the 35th (final) day after the insertion surgery predicted by the current DLN and mechano-regulatory method (referred as ground truth) are shown in Fig. 3. Implant A was with two different thread depths, resulting in severe bone resorption occurred around the threads in the lower part of the implant. Implant B was with the eagle-beak design proposed by Li et al.^17^. The implant C and D were commercial ITI solid cylindrical screwed implants (number 033.502S and 033.512S, Institute Straumann AG, Waldenburg, Switzerland). It is worth noting that Implants C was a dental implant used in the experimental study done by Marin et al.^18^, where our predicted bone resorption area has very good agreement with the experimental observation. For Implant D, there was merely no resorption around the implant body. However, cartilage almost covered the implant, and most of the surrounding bones were immature.

**Figure 4:**
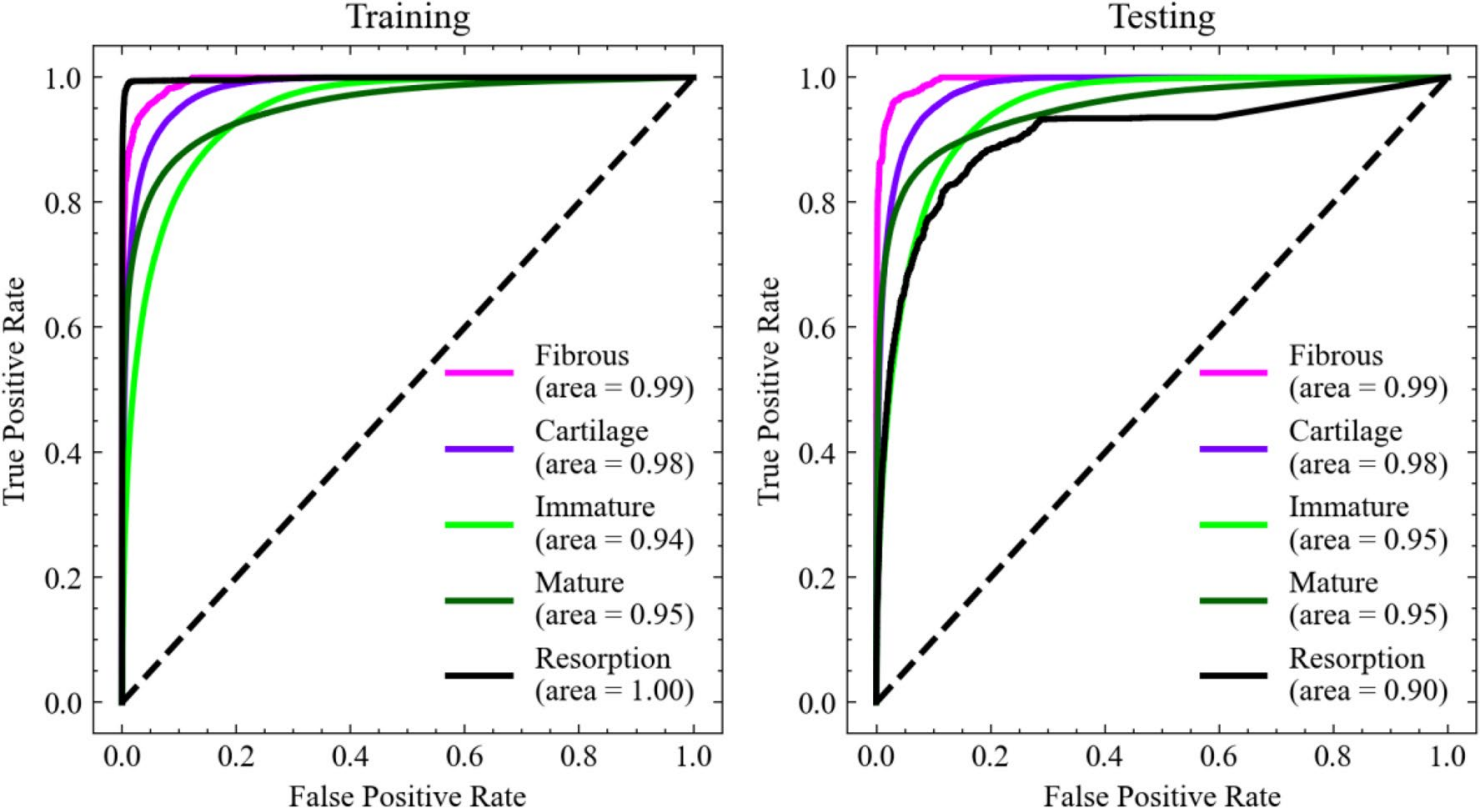
ROC curves for the classification of each tissue phenotype.

Figure 3 shows the distribution of tissue phenotype predicted by the current DLN and the ground truth generated by mechano-regulatory method. Also, figures of misclassification are shown, illustrating the pixels in dark red, where their tissue phenotypes predicted by the two methods were different. The pixels, where misclassified by the DLN, mainly accumulated at the first thread and the region underneath of the implant. It can be observed that the results predicted by the DLN have very good agreement with ground truths. It is remarkable that, the DLN predicted results were simply based on the input data on the 0th day. Surprisingly, the accumulation of error after 35 successive time-dependent iterations was not significant. The pixels which were accurately predicted were in average 88% of all the pixels in the region of interest (implant is not included) in the entire dataset. The corresponding distribution of the accuracy of the predicted tissue phenotypes for all the 65 cases are shown in the supplementary. Moreover, the performance of the current DLN was also evaluated by the receiver operating characteristic (ROC) curves and calculating the corresponding area under curves (AUC). Figure 4 shows the ROC curves for the classification of each tissue phenotype on every iteration (day) around the implants in the training and testing datasets. It shows that the DLN can accurately predict the differentiated tissue phenotype with testing AUC values of 0.99, 0.98, 0.95, 0.95, and 0.90 for fibrous tissue, cartilage, immature bone, mature bone, and resorption, respectively. The trained DLN showed robust performance for classifying each tissue phenotype.

**Figure 5:**
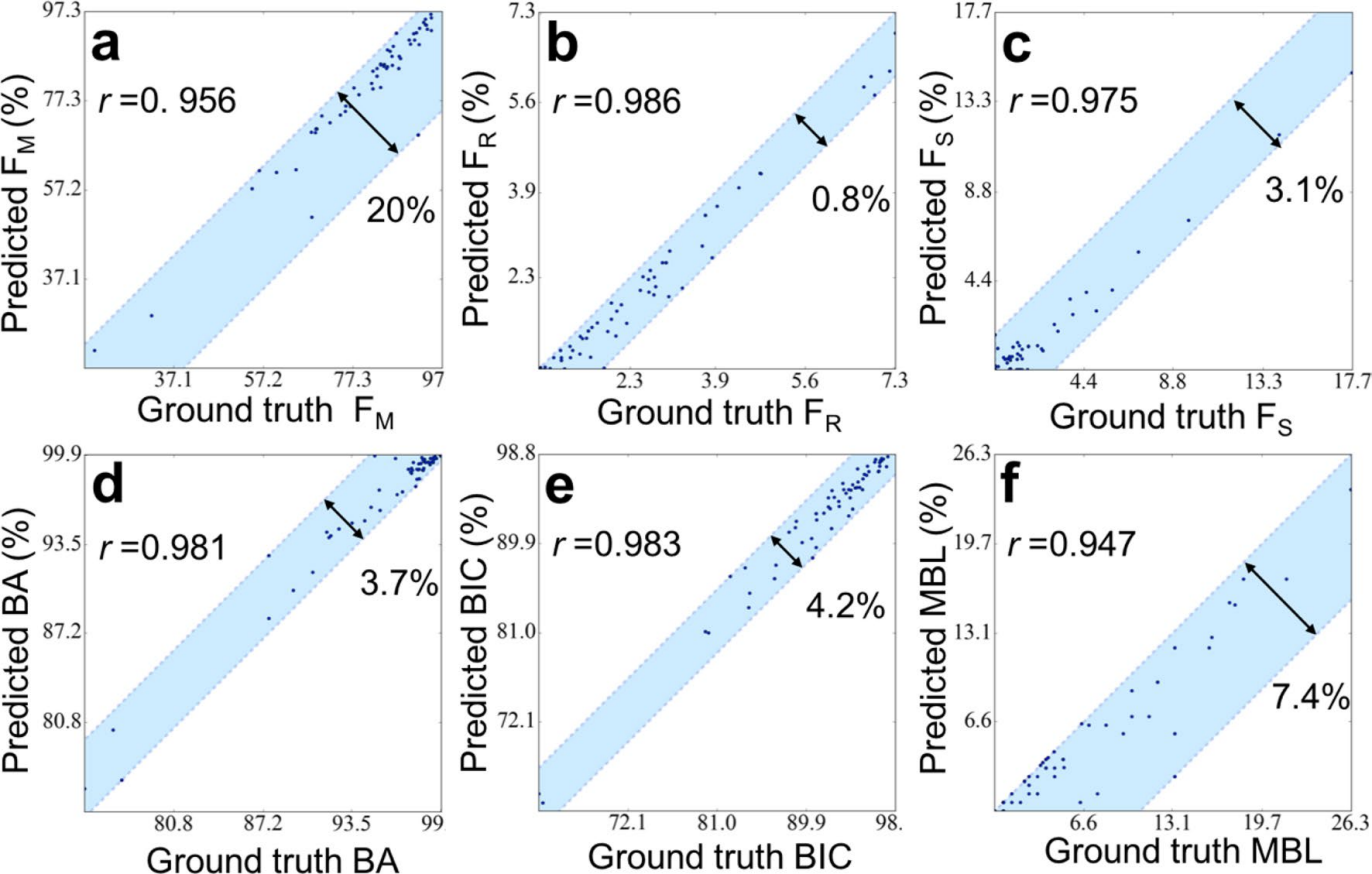
The predicted values vs. ground truth of six performance index (the percentage of mature bone (F_M_), bone resorption (F_R_) and soft tissue (F_S_), bone area (BA), bone-implant-contact (BIC), and marginal bone loss (MBL)) for all the 65 dental implants.

Now consider six performance indexes for the 65 dental implants based on the tissue phenotypes predicted on the 35th day. These indexes were commonly used in the study of dental implants, i.e., the percentage of mature bone (F_M_), bone resorption (F_R_) and soft tissue (F_S_), bone area (BA), bone-implant-contact (BIC) and marginal bone loss (MBL, i.e. bone resorption above the first thread). Where the region of interest of BA, BIC, and MBL are illustrated in the supplementary (Fig. S3c,d). Note that these indexes are high-level metrics, which were not directly predicted by the current DL, and further post-image processing was required. The predicted values vs. ground truth for all 65 implants are shown in Fig. 5. The light blue region contains all the 65 data points; the double arrows indicate the width of the blue region. It can be observed that the data points in the plots of F_R_ and MBL were rather underestimated by the current DLN. It is due to the problems of data imbalance of the resorption type and the ambiguity between the resorption and mature bones, as mentioned above. This also resulted in some of the pixels were misclassified as mature bone, and thus data points in the BA plot were overestimated. Even though it can be observed that Pearson correlation coefficients of all the indexes were greater than 0.947. It is remarkable that even the loss functions used for training were in a pixel-wise sense, and these six performance indexes which are the statistical information of the entire image can still be accurately predicted.

## Discussion

### Performance of the current DLN

**Figure 6:**
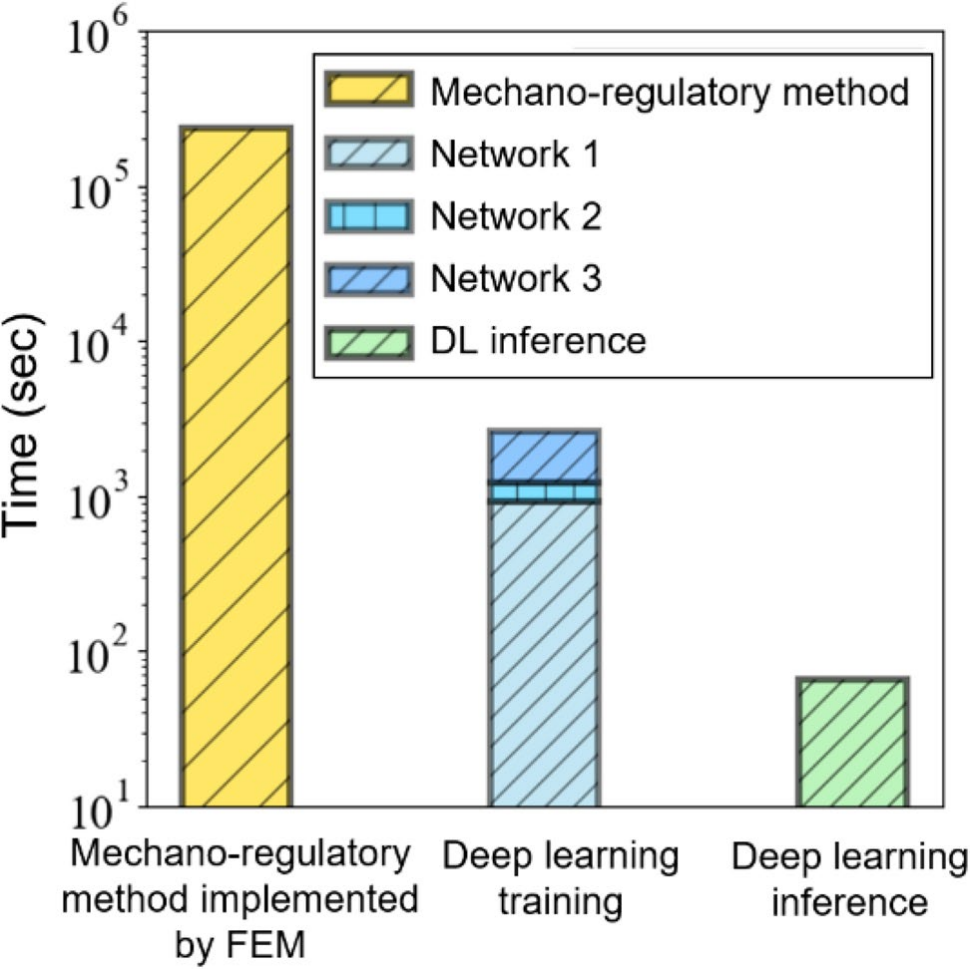
The comparison of computational resource consumption between mechano-regulatory method implemented by FEM and DLN.

Based on the results compared between the DLN and mechano-regulatory method implemented by FEM. The DLN proved its applicability to be an alternative to FEM and a highly-complex biomechanical model. Figure 6 reveals the computational resource consumption of mechano-regulatory method and the DLN in this study. The total computing time of the current DLN (including training time and inference time) was two orders fewer compared to that of mechano-regulatory method. If we only took into account single inference, the computing time reduction was in three orders of magnitude. It is worth mentioning that it is impossible to obtain a perfect DLN with a finite dataset. As feeding more simulation datasets to the current network, the accuracy of the prediction improves. This DLN has demonstrated a rapid preoperative evaluation of dental implants. It is expected to serve as a screening system eliminating implants with pool design, and to save a significant amount of time and costs on implant design.

### From black box to interpretable

In order to make the DLN interpretable, Deep Taylor decomposition^19^ (DTD) was applied. It redistributed the relevance between input data and the predicted results layer by layer, enabling the feature explanation for each implant case. This decomposition analysis provided in-depth insight on the relationship between (1) the physical properties and tissue phenotypes; (2) the geometry of implants and tissue phenotypes. They are discussed as follows.

### The relevance between physical properties and tissue phenotypes

**Figure 7:**
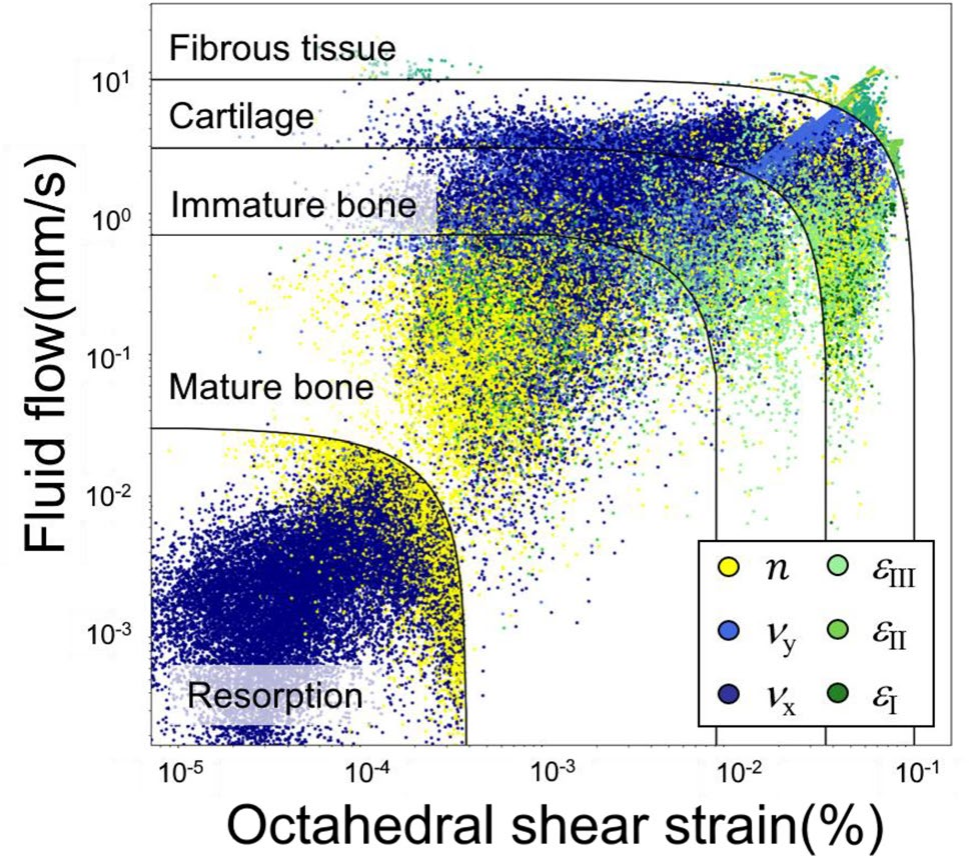
The phase diagram of tissue transition regions (from fibrous tissue to resorption) with logarithmic axes of fluid flow and octahedral shear strain. The color of each point indicates the most important physical property among the six to the corresponding tissue region provided by DTD.

Figure 7 is a phase diagram of tissue phenotypes with logarithmic axes, showing regions of fibrous tissue to resorption corresponding to magnitudes of the two bio-stimuli: octahedral shear strain *γ* and fluid velocity *ν*. The data points included all the pixels throughout 35 days in the 14 implants used in Figs. 2 and 3. The regions separated by black lines in Fig. 7 represent the five tissue phenotypes. The color of each data point indicates the most important physical properties among the six based on the results of DTD. Note that, for illustration purposes, only a quarter of the data points chosen randomly are shown. It seems to be obvious that the properties related to *ν* should dominate in the upper left triangular region, while the properties related to *γ* should dominate in the lower right triangular region. This statement is true for cartilage and immature bone only, i.e. dark blue points are typically located higher than green points in Fig. 7.

However, in the regions of mature bone and resorption do not follow that statement, and are dominated by concentration *n* and *ν*_*x*_ (the x-component of *ν*), respectively. This statement makes sense for mature bones, as they dominate in the final stage of the healing period with high stem cell concentration. Next, in order to verify the correlation between *ν*_*x*_ and resorption suggested by DTD, we have examined the distribution of *ν*_*x*_ around all the 65 implants on the 35th day. We then discovered that, regions between threads have no resorption as long as an alternating positive and negative *ν*_*x*_ pattern occurred in that region. Examples of *ν*_*x*_ with such alternating pattern can be found in Implants A (upper half), B, and D as shown in Fig. 3, where less resorption occurred at the corresponding location. The results proved that the current DLN is capable to capture the hidden attributions and provide physical insights without professional supervision.

### The relevance between geometry of implants and tissue phenotypes

**Figure 8:**
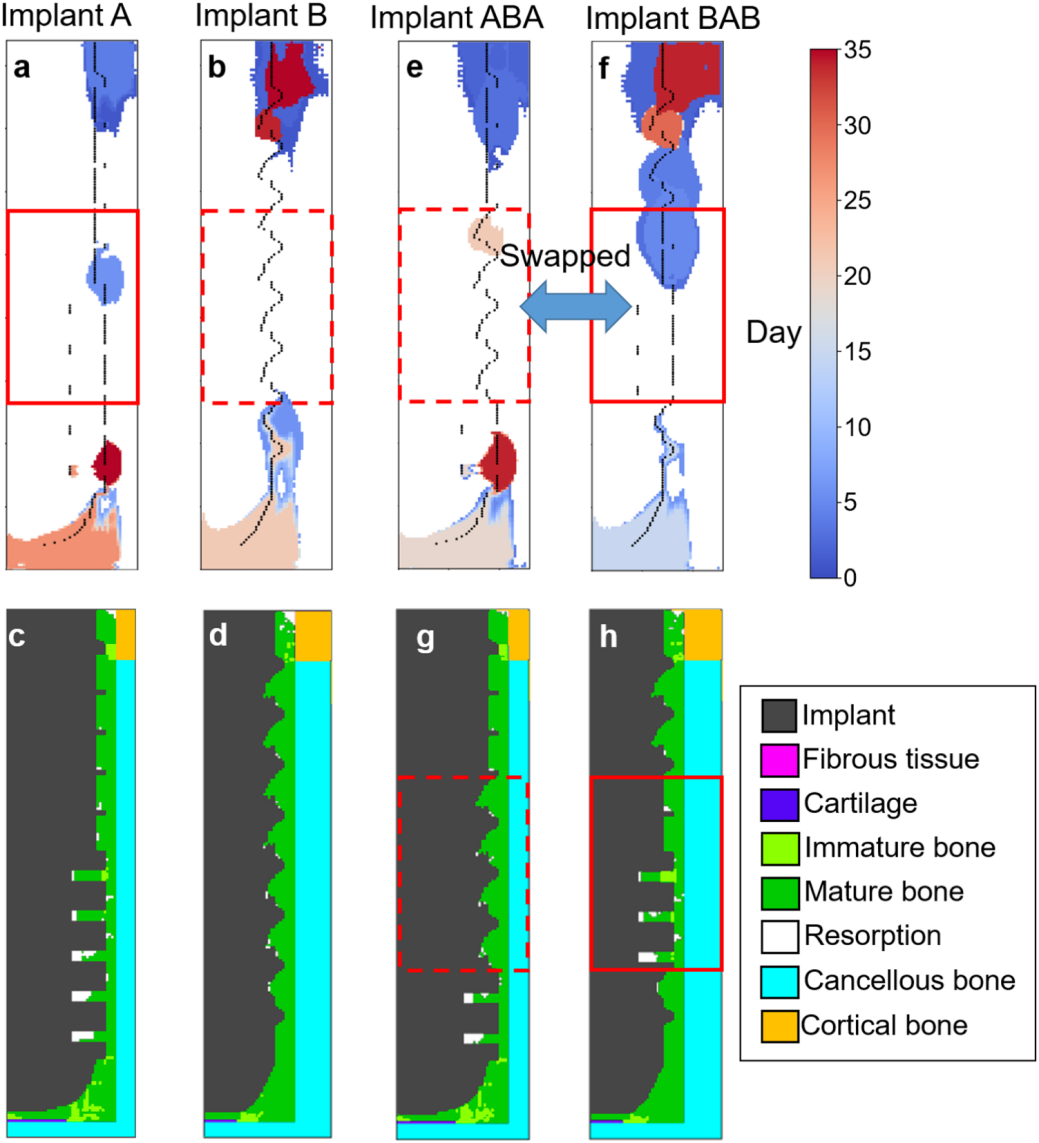
**(a,b,e,f)** The most important pixels of Implants A, B, ABA, and BAB for the overall distribution of tissue phenotype are colored corresponding to the 0th to 35th day (blue to red), suggested by DTD. **(c,d,g,h)** The distribution of tissue phenotype of the four implants on the 35th day, showing that swapping of the middle part of Implants A and B leads no significant effect on the overall distribution of tissue phenotype.

DTD also provides the pixel-wise relevance score to output prediction. In other words, the critical parts of the geometry of implants, which affect the resulting tissue phenotype the most, can be revealed. Two examples, Implants A and B, are shown in Fig. 8a,b. Where the black lines outline the shape of implants; the most important pixels are colored corresponding to the iterations, i.e. 0 to 35 days (blue to red). The corresponding distribution of tissue phenotype are shown in Fig. 8c,d where severe bone resorption occurred in Implant A and bone resorption was suppressed in Implant B. In the case of Implant A, pixels in the upper region were essential in the first few iterations and then moved toward the lower regions after the 20th day. On the other hand, an alternating pattern, i.e., the upper regions were essential at the early and final stages of iterations, was observed in the case of Implant B. The results indicated that the upper and lower parts of implants have higher influences on the overall distribution of tissue phenotype.

To verify this statement, the middle parts of Implants A and B (red squares with solid and dashed lines) were swapped, resulting in two new implants labeled as Implants ABA and BAB, as shown in Fig. 8e,f, respectively. The predicted distributions of tissue phenotypes shown in Fig. 8g,h. Interestingly, the position of the pixels of importance in the cases of Implants A and ABA are very similar, even though the design of the middle parts are significantly different, as shown in Fig. 8a,e. Next, Fig. 8b,f show that the alternating pattern of pixels of importance discovered in Implants B is also found in Implant BAB. Moreover, the distribution of regions outside the red squares in Fig. 8c,d are very similar to Fig. 8g,h, respectively. In other words, the swapping of the middle parts only affects the local cell differentiation within the red squares, and has almost no effect on the remaining parts of the bones.

It is striking that the current DLN, with no prior professional knowledge in dental clinical expertise, provided design guideline for dental implants that designers are advised to put more focus on the upper and lower parts of implants, instead of the middle part, to give a more effective influence on the overall bone healing. This statement matches the commercial design strategies that most of special designs focus on the upper or lower parts of implants. Many examples can be found in the market of dental implants: NeO Implants^20^ (Alpha Bio Tech, Petah Tykva, Israel) with cutting flutes at both the upper and lower parts; Laser-Lok implants^21,22^ (BioHorizons, Birmingham, England) with microchannels at the upper part; NobelSpeedy implants^23^ (NobelSpeedy Groovy, Nobel Biocare AB, Gothenburg, Sweden) with sharp apex cuts at the lower part; Endopore Hybrid implants^24,25^ (Endopore Dental System, Innova Corporation, Toronto, ON, Canada) with no threads apart from the upper part. The current DLN successfully extracted such important features mentioned above, purely based on the dataset of 65 implants generated by mechano-regulatory method with the training time less than several hours. The current DLN has great potential to be beneficial to dental implant industries.

### Discussion about the choices made by the current work

There are two major choices made here, i.e. the attribution technique and the applied design example. As for the attribution technique, there exists other explainable neural networks such as GNN (Graph Convolutional Neural Networks), which is particularly powerful in explaining the classification problems of molecular structures^26^. However, this type of graph problems may not cover the most of the common engineering design process, such as device design or multiscale physics. In addition, the workflow used in the present work is implemented by ANN and U-net, which are suitable to be explained by Deep Taylor Decomposition, Guided Backpropagation and Gradient-based Saliency Maps according to the literature^27,28^. Thus, we have applied the three techniques on Implants A and B in the manuscript to benchmark the performance of explainability, as shown in Fig. S5 in Supplementary. The results show that DTD can catch the critical parts of implant in the respect of design of geometry. Thus, DTD is adopted as our relevance analysis in this work.

Next, the current dental implant design problem is a representative example to demonstrate the generality of the current deep learning network. As for the functionality of DLNs, the current work contains both regression and classification problems. Where Network 1 as a surrogate model for FEM part is the former, while the Network s 2 and 3 as tissue type predictors are the later. As for the complexity of the current problem, mechano-regulatory algorithm contains the mechanical-fluid coupled and diffusion calculations with gradient material properties varying with time iteratively. It is expected that such complexity covers most of the calculations in the common engineering design process.

## Conclusion

In the current study, a deep learning network which can instantly evaluate the performance of dental implants before surgeries is developed. The training dataset included 65 dental implants and the corresponding distribution of tissue phenotypes from the 0th to 35th days, generated by mechano-regulatory method. The essential physical properties, such as principle strains, fluid velocities, and stem cell concentration, of surrounding bones were accurately predicted (the correlation coefficients *r* > 0.980). These properties normally require hours of FEM calculation, and now can be predicted within seconds. The performance indexes, such as the percentage of bone area and bone-implant-contact, and marginal bone loss, of implants were also predicted (*r* > 0.947).

This level of accuracy is achieved by tailor-made DLN design, consisting of three networks. Network 1, modified from U-Net and Inception block, was used to replace FEM simulation procedures. Network 2 (ANN) was used to classify the tissue phenotype. Network 3, modified from U-Net, was used to de-noise and reduce accumulation of error throughout the iterations. Also, focal loss function and random forest algorithm were used to deal with the problems of data imbalance and type II error of the resorption type. The network design enables predicting results of 35 days with data on the 0th day as input data only.

Moreover, the success of the DLN is its interpretability. Based on the results obtained via Deep Taylor decomposition, it is suggested that the transverse fluid velocity, upper and lower parts of dental implants are the keys that influence bone healing and the distribution of tissue phenotypes the most. Many examples of commercial dental implants with designs which follow these design strategies can be found. This proves that the design guidelines provided by the current DLN without prior knowledge of dental clinics, match the experiences learned in the dental implant industries for years. This work is a proof of concept that deep learning approach can be an alternative to complex, time-dependent, multi-physical models/theories, such as mechano-regulatory method, as well as to reveal the underlying features without clinical expertise.

## Methods

### Mechano-regulatory method overview

Mechano-regulatory method is a well-accepted algorithm to predict the history of bone healing. The algorithm firstly determined the local octahedral shear strain *γ* and relative fluid/solid velocity *ν* of the region around the implants subjected to the given load. Then, the local tissue phenotype on the next iteration can be determined by the cell stimulus factor *S*

1 $$S = \gamma /a + \nu /b$$

where *a* = 0.0375 and fluid stimulus constant *b* = 3 μm/s^10^. The corresponding range of the value of *S* for these tissue phenotypes are listed in Table S1^29^ in the supplementary. Each tissue phenotype has a set of material properties^30–34^, as shown in Table S2 in the supplementary. Note that the effective material properties for the next iteration is the linear combination between the material properties of the granulation tissue *X*_*g*_ initially filled in the callus region and the differentiated tissue phenotype *X*_*d*_ based on the concentration of the stem cell *n* diffused from the boundary of the cell-differentiation region, such that

2 $$X=\frac{{n}^{max}-n}{{n}^{max}}{X}_{g}+\frac{n}{{n}^{max}}{X}_{d}$$

where ${n}^{max}$ is the maximum concentration. In addition, the smoothing procedure was applied by averaging the properties with the those in the previous nine steps to avoid the numerical instability^29^. The detailed workflow of mechano-regulatory method can be found in the supplementary (Fig. S1).

### Data preparation by FEM simulation

Mechano-regulatory method was implemented by FEM package ANSYS. Simulation data of 65 dental implants with different geometries and the corresponding bone healing history throughout 35 days was generated by ANSYS and Matlab. Structured rectangular meshes were adopted in the current model, allowing a direct transfer between elements and pixels without any interpolation. The two-dimensional axisymmetric FE model included a Ti-6Al-4 V implant, a cortical bone layer, cancellous bones, and calluses. The implant and the remaining parts were meshed by PLANE42 and CPT212 (coupled pore-pressure mechanical element) provided by ANSYS. Biting load was applied via a displacement of 8 μm on the top of the implant. The axisymmetric boundary condition and constraints of nodes were shown in Supplementary Fig. S3a. The callus region located around the implant was the cell-differentiation regions. One of its boundaries, marked as the dashed line, was the cells origin, which is the source of the diffusion of stem cells.

### Network setting and training

The hyper-parameters of Network I–III were optimized by Bayesian method. The number of filters, corresponding filter size and activation function for network I and III (U-net) and the amount of units in each dense layer, number of layers, and suitable regularization weight for Network II (ANN) were determined after 50 trials of network training. Each training includes 50 epochs.

After the structures of the networks were set, Adam optimization algorithm was applied^35^. The dataset was then split into a twenty percent testing set and an eighty percent training set. During the training process, the early stopping method was applied where the parameter of patience was set as 20 epochs. Next, for each case of dental implant, dataset of 35 images corresponding to 35 days of the distribution of tissue phenotypes were recomposed into 630 pairs of input and output images as training dataset, i.e. Day 0 and 1, Day 0 and 2, …, Day 0 and 35, Day 1 and 2, Day 1 to 3, …, Day 33 to 35, Day 34 to 35. This training strategy allowed the DLN establishes the features relationship between every successive day, reducing error accumulation for long-term predictions. After the training, the resulting DLN achieved in average 88% accuracy of tissue phenotype prediction on Day 35 in the cell-differentiation regions, for both the training and testing sets with the image of Day 0 as the input data only. High testing AUCs (ranging from 0.90 to 0.99) is also achieved. Note that the current problem is related to bone healing and mechano-regulatory algorithm which are time dependent and highly complicated. It typically requires large amount of dataset. However, it is remarkable that the current network design and the training strategy enable highly-accurate long-term predictions based on short-term training and limited dataset (65 dental implants only).

## Supplementary Information

Supplementary Information.
