# Supplementary material for: Engineer design process assisted by explainable deep learning network
Source: Sci Rep. 2021 Nov 18;11:22525. doi: 10.1038/s41598-021-01937-5 (PMC8602721; doi:10.1038/s41598-021-01937-5)
Supplement: Supplementary file 1 — Supplementary Information. [file 41598_2021_1937_MOESM1_ESM.docx]

**Supplementary**

**The reasons why the current work chose a multi-model pipeline over a single End-to-End model.** We did try a single End-to-End (E2E) network as a tissue phenotype classifier in the early stage of this work. However, there are several reasons that make it an infeasible option in this study. First, the values of two biophysical stimuli: strain and fluid velocity that corelate with tissue differentiation are in range of 4 to 6 orders as shown in Fig. 7 in the manuscript. It is difficult to retain accuracy and generality in an E2E model simultaneously. Second, it is more difficult for the researchers to correlate the most influential factors extract by DTD with physical properties from a single E2E model than a multi-model pipeline where each of models has clear functionality. Third, the accuracy of tissue type predictor is limited if an E2E model is used. This is because the tissue type of each pixel is greatly influenced by its surrounding and position in the entire image.

To solve the issues mentioned above, we developed three networks with clear functionality for the explainability of the current deep learning network. Where network 1 focuses on FEM calculation; This design allows DTD to attribute the most influential factors and to correlate them with the results of FEM, such as strain and fluid velocity components. Network 2 is used to give an estimation of the tissue type for each pixel based on the local properties predicted by Network 1. It is expected that great discontinuity of the predicted tissue type between elements may be generated by Network 2 as the position and neighbor information are not considered. Then, Network 3 is trained to calibrate the unreasonable predictions made by Network 2 by taking the position information in the image into account. The design solve all the three issues mentioned in the previous paragraph.

**Deep learning framework structure and mechano-regulatory method workflow.** Figure S1 shows the workflow of mechano-regulatory method. The loop went through 35 times for each case of dental implant, to obtain 35-days bone healing history. The part of FEM calculation was colored in yellow. The part of classifier of tissue phenotype was colored in Blue. Note that the range of the value of cell stimulus factor *S* corresponding to the five tissue phenotypes were listed in Table S1. Figure S2 shows the workflow of the current deep learning framework for comparisons.


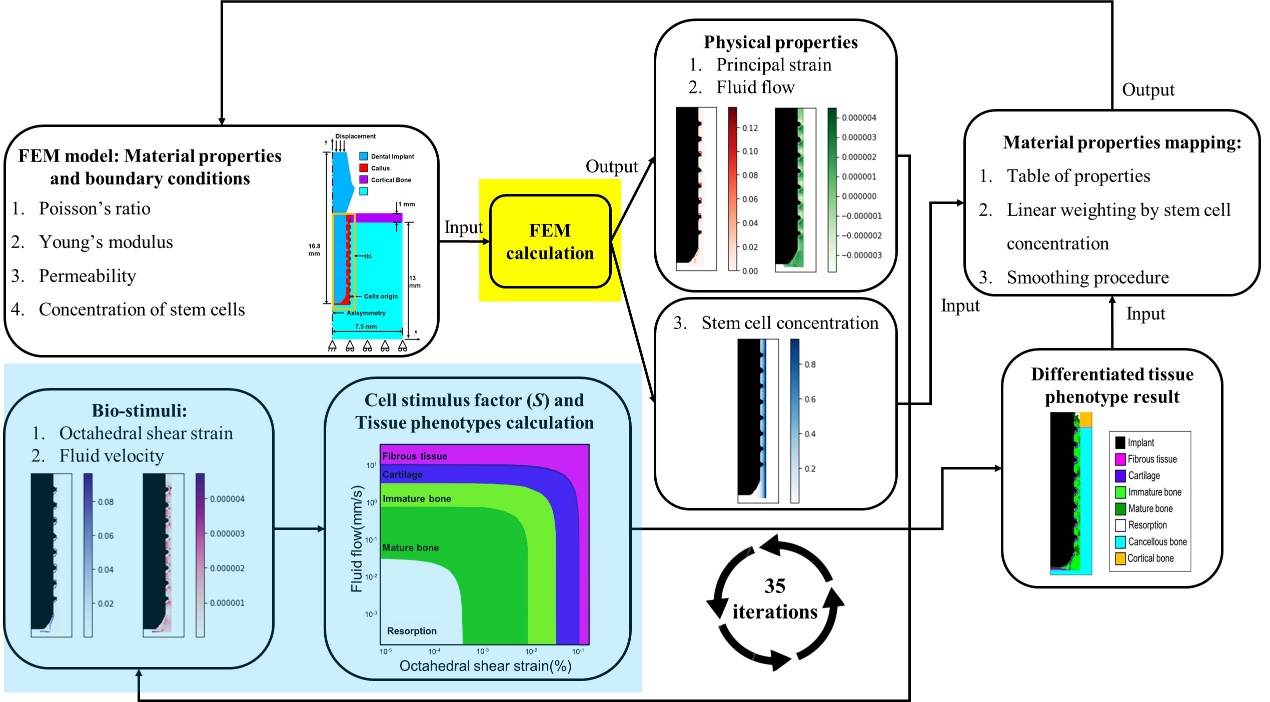


Fig. S1 The workflow of mechano-regulatory method.


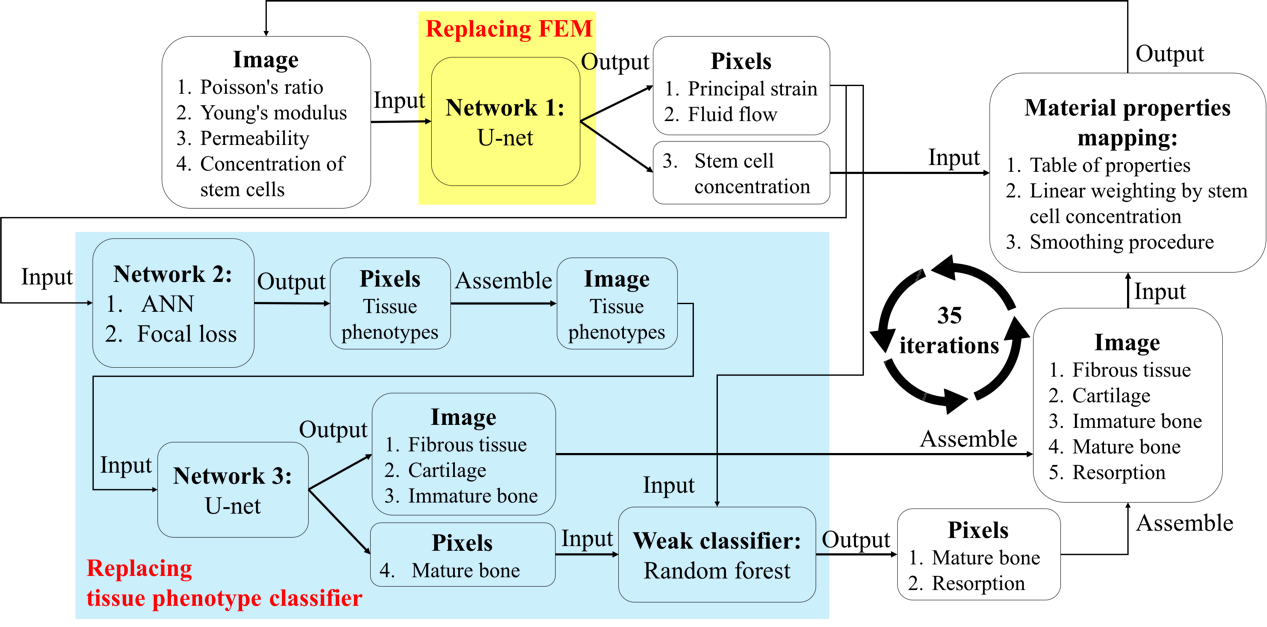


Fig. S2 The current DL framework structure.

Table S1 The ranges of cell stimulus factor for different tissue phenotypes.

|  | *S* |  | Tissue phenotype |
| --- | --- | --- | --- |
| 3< | *S* |  | Fibrous tissue |
| 1< | *S* | $\leq$3 | Cartilage |
| 0.266< | *S* | $\leq$1 | Immature bone |
| 0.010< | *S* | $\leq$0.266 | Mature bone |
|  | *S* | $\leq$0.010 | Initial resorption |

**Finite element model setting.** The geometry of the dental implant model and the applied boundary conditions in FEM calculation are shown in Fig. S3 a. The model is two-dimensional axisymmetric about the y axis. A downward displacement of 8um along the y axis corresponding to a biting force of 100N was applied on the top of the implant. The node at the bottom-left was fixed, while the remaining nodes at the bottom were constrained in the y axis. The material properties of Ti-6Al-4V dental implants, tissue phenotypes, and bones used in the current FEM calculation are listed in Table S2.

The dashed line in Fig. S3 a indicates cell origin which is defined as the source of stem cell migration. The area marked by the yellow rectangle in Fig. S3 a is the region as input data for machine learning. It was meshed by 210 × 52 elements allowing a direct transformation into pixels. An enlarged image as input data are shown in Fig. S3 b.

Bone-implant contact (BIC), bone area (BA) and marginal bone loss (MBL) were used in the current study to evaluating the performance of dental implants. MBL is defined as the percentage of the total area of resorption elements in the region of interest (ROI). ROI for MBL is the total area above the first thread (illustrated as shaded area) in Fig. S3 c. BIC is defined as the percentage of the total length of the interface between the implant elements and the mature/immature bone elements in ROI of BIC. Where the ROI of BIC is the total length of all the threads in the implant. ROI of BIC in one of the threads is illustrated with a red solid line in Fig. S3 d. Similarly, BA is the percentage of the total area of the mature/immature bone elements in the ROI of BA. ROI of BA is the total area between all the threads (illustrated as shaded area) in Fig. S3 d.

Table S2 Material properties of the tissues used in the current model.

|  | Young’s modulus  (MPa) | Poisson’s ratio | Permeability (m^4^/Ns) |
| --- | --- | --- | --- |
| Granulation tissue | 1 | 0.17 | 10^-14^ |
| Fibrous tissue | 2 | 0.17 | 10^-14^ |
| Cartilage | 10 | 0.17 | 5$\times$10^-15^ |
| Immature bone | 1000 | 0.30 | 10^-13^ |
| Mature bone | 6000 | 0.30 | 3.7$\times$10^-13^ |
| Cortical bone | 20000 | 0.30 | 10^-17^ |
| Cancellous bone | 6000 | 0.30 | 3.7$\times$10^-13^ |
| Ti-6Al-4V | 113000 | 0.30 | N/A |


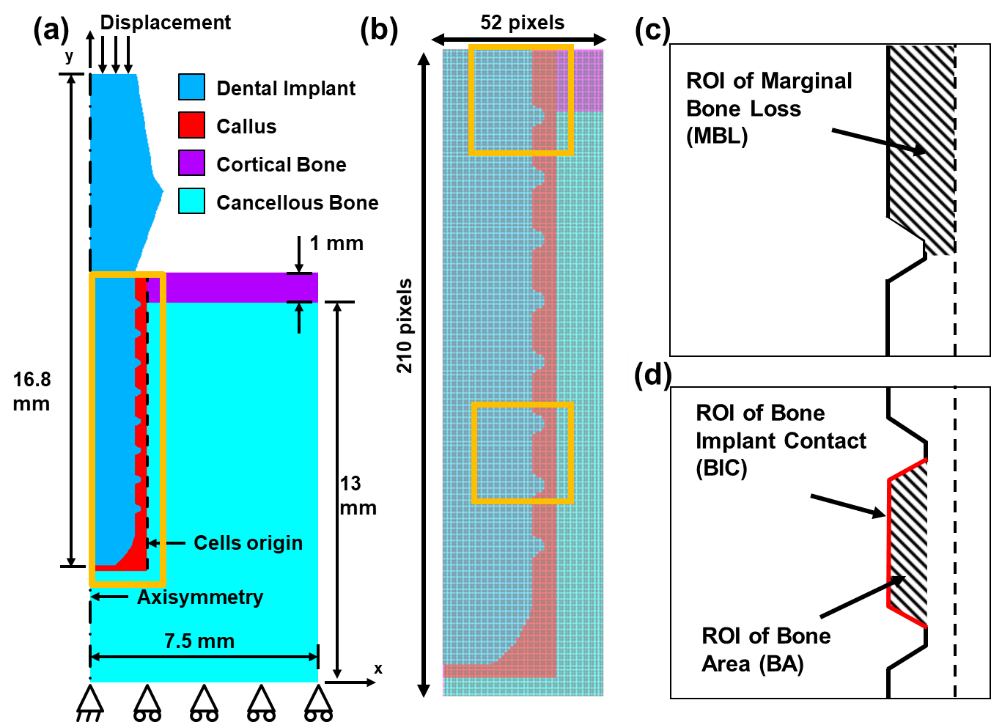


Fig. S3 (a) The illustration of the FEM model for dental implant and bones. (b) The region marked by the yellow rectangle in (a), which is a typical input image data for machine learning. (c) ROI of MBL. (d) ROI of BIC and BA in one thread.

**The distribution of the accuracy of the predicted tissue phenotypes.** Fig. S4 shows a histogram plot of the accuracy of the predicted tissue phenotypes on the 35th day in the cell-differentiation regions, i.e. the red region shown in Fig. S3 a, of all the 65 cases of dental implant. It is remarkable that, even after a complete healing period (i.e. 35 days/iterations), the mean accuracy 88% was achieved.


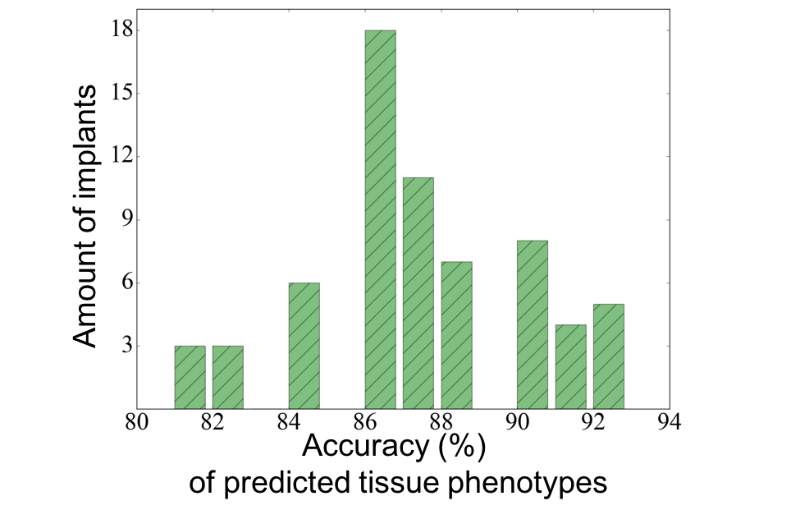


Fig. S4 The histogram plot of the accuracy of the predicted tissue phenotypes on the 35^th^ day in the cell-differentiation regions of all the 65 cases of dental implant.

**The choice for attribution technique.** There exists other explainable neural networks such as GNN (Graph Convolutional Neural Networks), which is particularly powerful in explainability for the classification problems of molecular structures^1^. However, this type of graph problems may not cover the most of the common engineering design process, such as device design or multiscale physics. In addition, the workflow used in the present work is implemented by ANN and U-net, which are suitable to be explained by Deep Taylor Decomposition, Guided Backpropagation and Gradient-based Saliency Maps according to the literature^2,3^.

Thus, we have applied the three techniques on Implants A and B in the manuscript to benchmark the performance of explainability, as shown in Fig. S5. It can be observed that similar trends are generated by the three methods. However, DTD pays more attention on the surface of implants, i.e. the design geometry, while the others pay more attention on the inner regions of implants. This indicates that DTD can catch the critical parts of implant in the respect of design of geometry. Thus, DTD is adopted as our relevance analysis in this work.


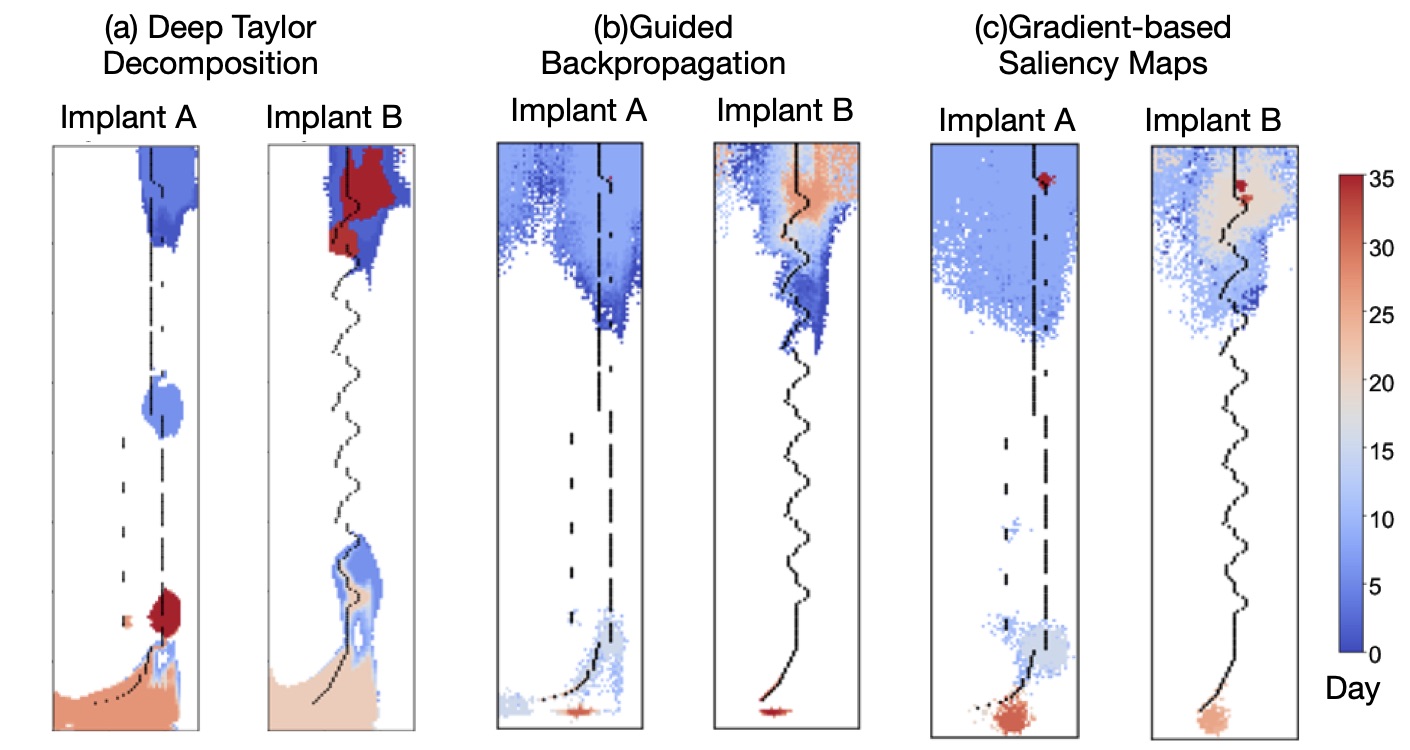


Fig S5. The comparison between interpretability results by (a) Deep Taylor Decomposition, (b) Guided Backpropagation, and (c)Gradient-based Saliency Maps.

1. Pope, P. E., Kolouri, S., Rostami, M., Martin, C. E. &Hoffmann, H. Explainability methods for graph convolutional neural networks. in *Proceedings of the IEEE Computer Society Conference on Computer Vision and Pattern Recognition* vols 2019-June (2019).
2. Simonyan, K., Vedaldi, A. &Zisserman, A. Deep inside convolutional networks: Visualising image classification models and saliency maps. in *2nd International Conference on Learning Representations, ICLR 2014 - Workshop Track Proceedings* (2014).
3. Springenberg, J. T., Dosovitskiy, A., Brox, T. &Riedmiller, M. Striving for simplicity: The all convolutional net. in *3rd International Conference on Learning Representations, ICLR 2015 - Workshop Track Proceedings* (2015).
